# GAD-alum immunotherapy in type 1 diabetes expands bifunctional Th1/Th2 autoreactive CD4 T cells

**DOI:** 10.1007/s00125-020-05130-7

**Published:** 2020-04-04

**Authors:** Sefina Arif, Iria Gomez-Tourino, Yogesh Kamra, Irma Pujol-Autonell, Emily Hanton, Timothy Tree, Daisy Melandri, Caroline Hull, Diane K. Wherrett, Craig Beam, Bart O. Roep, Anna Lorenc, Mark Peakman

**Affiliations:** 1 Peter Gorer Department of Immunobiology, King’s College London Faculty of Life Sciences and Medicine, 2nd Floor, Borough Wing, Guy’s Hospital, London, SE1 9RT UK; 2 Division of Endocrinology, Department of Pediatrics, The Hospital for Sick Children, University of Toronto, Toronto, ON Canada; 3 Homer Stryker MD School of Medicine, Western Michigan University, Kalamazoo, MI USA; 4 Diabetes and Metabolism Research Institute, City of Hope, Duarte, CA USA; 5 King’s Health Partners Institute of Diabetes, Endocrinology and Obesity, King’s College Hospital NHS Foundation Trust, London, UK

**Keywords:** Autoreactive, Epitopes, GAD, GAD-alum, Glutamic acid decarboxylase, IL-13, Immunotherapy, T cell receptor, T cells, TCR, Th2, TrialNet

## Abstract

**Aims/hypothesis:**

Antigen-specific therapy aims to modify inflammatory T cell responses in type 1 diabetes and restore immune tolerance. One strategy employs GAD65 conjugated to aluminium hydroxide (GAD-alum) to take advantage of the T helper (Th)2-biasing adjuvant properties of alum and thereby regulate pathological Th1 autoimmunity. We explored the cellular and molecular mechanism of GAD-alum action in the setting of a previously reported randomised placebo-controlled clinical trial conducted by Type 1 Diabetes TrialNet.

**Methods:**

In the clinical trial conducted by Type 1 Diabetes TrialNet, participants were immunised with 20 μg GAD-alum (twice or three times) or alum alone and peripheral blood mononuclear cell samples were banked at baseline and post treatment. In the present study, GAD-specific T cell responses were measured in these samples and GAD-specific T cell lines and clones were generated, which were then further characterised.

**Results:**

At day 91 post immunisation, we detected GAD-specific IL-13^+^ CD4 T cell responses significantly more frequently in participants immunised with GAD-alum (71% and 94% treated twice or three times, respectively) compared with those immunised with alum alone (38%; *p* = 0.003 and *p* = 0.0002, respectively) accompanied by high secreted levels of IL-13, IL-4 and IL-5, confirming a GAD-specific, GAD-alum-induced Th2 response. Of note, GAD-specific, IL-13^+^ CD4 T cells observed after immunisation co-secreted IFN-γ, displaying a bifunctional Th1/Th2 phenotype. Single-cell transcriptome analysis identified *IL13* and *IFNG* expression in concert with the canonical Th2 and Th1 transcription factor genes *GATA3* and *TBX21*, respectively. T cell receptor β-chain (TCRB) CDR3 regions of GAD-specific bifunctional T cells were identified in circulating naive and central memory CD4 T cell pools of non-immunised participants with new-onset type 1 diabetes and healthy individuals, suggesting the potential for bifunctional responses to be generated de novo by GAD-alum immunisation or via expansion from an existing public repertoire.

**Conclusions/interpretation:**

GAD-alum immunisation activates and propagates GAD-specific CD4 T cells with a distinctive bifunctional phenotype, the functional analysis of which might be important in understanding therapeutic responses.

**Electronic supplementary material:**

The online version of this article (10.1007/s00125-020-05130-7) contains peer-reviewed but unedited supplementary material, which is available to authorised users.

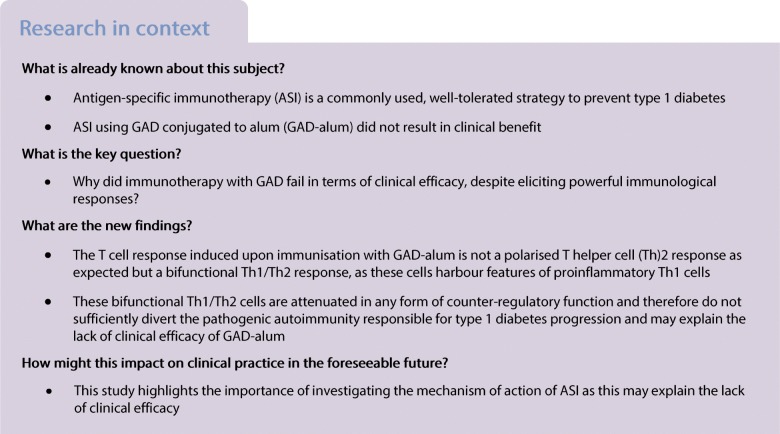



## Introduction

Type 1 diabetes develops in multiple stages preceded by immune cell activation and beta cell destruction. Stage 1 is characterised by the presence of autoantibodies targeting islet beta cell autoantigens, dysglycaemia is evident in stage 2 and stage 3 is characterised by symptomatic diabetes [1]. Therapeutic intervention to maintain beta cell function can be applied depending on whether the aim is prevention (prior to stage 1) or intervention (stages 1–3). Intervention studies include both antigen-specific and non-antigen-specific therapies.

Antigen-specific immunotherapy (ASI) has emerged as an attractive strategy for the prevention of type 1 diabetes development and loss of beta cell function. Based on successes in preclinical models, several early-stage clinical trials have been conducted using whole antigens presented orally, or antigens or peptides given by parenteral injection [2]. Although the results of these trials have been largely negative, most have been small and conducted at early stages of drug development on small numbers of individuals and have established that, according to current experience, ASI is very well tolerated and has a good safety profile [3].

One of the most advanced strategies in terms of drug development is the use of the autoantigen GAD65 conjugated to alum (GAD-alum) administered at low dose (20 μg) via the subcutaneous route. While an early Phase I/II study showed encouraging prevention of C-peptide decline in individuals with recent-onset type 1 diabetes, a Phase II study conducted by Type 1 Diabetes TrialNet did not find any indication of efficacy [4] and neither did a small study in high-risk, multiple-antibody-positive individuals [5]. Finally, a meta-analysis of published results in people with new-onset type 1 diabetes does not entirely rule out a therapeutic benefit from the GAD-alum injections [6], and therefore the question of the setting in which this approach might be used remains unresolved.

While clinical efficacy has not been demonstrated, there is little doubt that immunisation with GAD-alum has powerful immunological effects. A series of mechanistic studies accompanied the clinical trials conducted to date and demonstrated that GAD-alum injection is a potent inducer of high-titre anti-GAD65 autoantibodies and also results in changes in CD4 T lymphocyte activation, tissue homing receptor expression and cytokine release in response to stimulation with GAD65 in vitro [7, 8]. The overarching interpretation of these results is that the balance of CD4 T lymphocyte responses to GAD65 is shifted by therapy away from T helper (Th)1 and towards a Th2 response. Indeed, this was the original rationale for the approach of using alum as an adjuvant for GAD65, since it is known to bias CD4 T lymphocyte responses against co-administered antigens towards Th2 [9–11]. GAD-alum immunotherapy thus aimed to limit Th1-driven disease progression through the induction of autoantigen-specific Th2 responses, although evidence of this lacking.

These mechanistic outcomes present a conundrum, since while the immunological endpoints have largely been achieved in GAD-alum immunotherapy studies, this has not been accompanied by therapeutic benefit. In the present study we address the key question of whether the Th2 mechanistic rationale for GAD-alum immunotherapy is supported.

## Methods

### Participants

We studied 46 participants with type 1 diabetes recruited for the previously reported Type 1 Diabetes TrialNet TN08 study (‘Effects of Recombinant Human Glutamic Acid Decarboxylase (rhGAD65) Formulated in Alum (GAD-alum) on the Progression of Type 1 Diabetes in New Onset Subjects’, ClinicalTrials.gov no. NCT00529399) from 15 sites in the USA and Canada within 100 days of diagnosis; participants were diagnosed with type 1 diabetes according to American Diabetes Association criteria (Electronic supplementary material [ESM] Table 1) ([4]. The Type 1 Diabetes TrialNet TN08 study was a three-arm, multicentre, randomised, double-masked, placebo-controlled clinical trial designed to test the hypothesis that multiple injections of GAD-alum preserve endogenous insulin production in type 1 diabetes. The primary outcome was to establish whether C-peptide in the GAD-alum-treated group differed significantly from the placebo-treated group at 1 year.

At entry, all participants had GAD65 antibodies (GADA) and stimulated C-peptide concentrations of ≥0.2 nmol/l during a mixed-meal tolerance test (MMTT). Participants received three subcutaneous injections of GAD-alum or alum (Diamyd, Diamyd Medical, Stockholm, Sweden) at baseline (day 0), day 28 and day 56, as previously described [4]; specifically, 16 out of 46 participants received GAD-alum injections three times, 17 received GAD-alum twice and then one injection of alum and 13 received alum three times. Samples obtained at baseline and 3 months post treatment (i.e. 3 months after the baseline treatment) were banked and used in the present study, which aimed to address the lack of therapeutic benefit in this GAD-alum trial. Samples from three of the 46 participants (one treated with GAD-alum twice and two treated three times) together with a further individual (treated with GAD-alum three times; additional samples shipped by Trialnet, as we required more cells for the subsequent experiments) were used in subsequent T cell line generation and epitope-mapping studies. In addition to these participants, we studied GAD-specific IFN-γ responses in 71 non-trial individuals with recent-onset type 1 diabetes (47 men, mean age: 28.7 years, range: 18–46 years, duration of disease: 0.25–14.5 months) (ESM Table 2). These individuals were recruited via the ‘T cell studies in Type 1 diabetes’ study at Guy’s and St Thomas’ Hospital and Bristol Royal Infirmary. Informed consent was obtained from all participants and this study was approved by the UK National Research Ethics Committee.

### Detection of GAD-specific CD4 T cells

Cryopreserved peripheral blood mononuclear cells (PBMCs) from GAD-alum- and alum-immunised participants were thawed and stimulated with 10 μg/ml T cell GAD (recombinant human GAD expressed in baculovirus and formulated specifically for use in T cell assays) and GAD diluent (Diamyd Medical). Cultures were then examined for IL-13 responses by indirect ELISPOT (U-Cytech, Utrecht, Netherlands), as previously described [12]; in brief, IL-13 production by CD4 T cells was assessed by operators blinded to the immunisation status of the donor (GAD-alum or alum). Data are expressed as the mean number of spots per triplicate compared with the mean spot number in the presence of diluent alone (stimulation index [SI]). A receiver operating characteristic (ROC) plot was used to define optimal sensitivity and specificity and a cut-off of ≥2.5 defined as a positive response (sensitivity 73%, specificity 77%). Supernatants from PBMCs cultured with T cell GAD for 24 h during these ELISpot assays were assessed for the presence of IFN-γ, IL-13, IL-4, IL-5, IL-10, IL-17 and IL-6 by Luminex (Millipore UK, Watford, UK), with standard curves generated with a dynamic range between 3 and 10,000 pg/ml. Data were analysed using the Flexmap 3D software (R&D systems, Abingdon, UK).

In some assays, cytokine responses were measured using combined IL-13/IFN-γ indirect FluoroSpot assays (U-Cytech, Utrecht, the Netherlands) to examine cytokine co-secretion. Briefly, cells stimulated with GAD65, GAD peptide or the positive control (Pediacel, Sanofi, Guildford, Surrey) were transferred to wells of a pre-treated FluoroSpot plate coated with high affinity anti-IL-13 and anti-IFN-γ antibodies [12]. After 24 h the cells were removed by washing, and antibody-bound cytokine identified using a mixture of anti-IL-13 and anti-IFN-γ detection antibodies followed by two fluorescent conjugates containing Alexa 488-labelled anti-FITC antibodies and R-phycoerythrin (R-PE)-labelled-streptavidin made up according to manufacturer’s instructions. Green fluorescent spots represent IFN-γ producing cells, red spots, IL-13 or IL-4 and yellow spots signify cells that release both IFN-γ and IL-13/IL-4.

### T cell lines and clones

In order to generate GAD-specific T cell lines, cryopreserved PBMCs from participants (3 months post immunisation with GAD-alum) were thawed and stimulated with 10 μg/ml T cell GAD for 48 h; IL-13-secreting cells were captured using an IL-13 secretion/detection kit for 1 h at 37°C (Miltenyi Biotec, Bisley, Surrey, UK). Cells were subsequently labelled with mouse anti-human IL-13-biotin followed by anti-biotin PE in a cocktail containing monoclonal mouse anti-human CD3 APC-H7 (BD Biosciences Cat. No. 560275, RRID:AB_1645476), CD14 V500 (Cat. No. 541391), CD19 V500 (Cat. No. 561121), CD8 FITC (BD Biosciences Cat. No. 555634, RRID:AB_395996) (Becton Dickinson, Oxford, UK) and CD4 allophycocyanin (APC) (BioLegend Cat. No. 317415, RRID:AB_571944) (BioLegend, London, UK) all made up according to manufacturer’s instructions for 10 min on ice. Following a washing step, the cells were magnetically labelled with anti-PE microbeads, and a viability marker, 7-amino-actinomycin D (BioLegend, London, UK), was added. The cells were washed, and magnetic separation was performed using MS MACS columns (Miltenyi Biotec, Bisley, Surry, UK) to enrich for IL-13^+^ cells. CD4^+^IL-13^+^ T cells were then bulk sorted (typically 100–3000 cells were sorted per condition) using a FACSARIA II SORP cell sorter (Becton Dickinson) into polypropylene tubes containing 10^5^ irradiated mixed allogeneic PBMCs with 4 μg/ml phytohaemagglutinin (PHA) (Alere, Zug, Switzerland) in X-vivo-15 (Lonza, Slough, UK) containing 5% (wt/vol.) human AB serum (Sigma Aldrich, Gillingham, Dorset, UK). The next day, fresh medium supplemented with T cell growth factor (TCGF; final concentration 5% (wt/vol.); Cellkine, ZeptoMetrix, Buffalo, NY, USA) was added and cells were expanded in culture medium containing decreasing concentrations of TCGF for 2 weeks before testing for specificity by IL-13 ELISpot. In brief, GAD specificity was examined by direct IL-13 ELISpot (U-Cytech, Utrecht, the Netherlands) using 10^4^ T cells in the presence of 2×10^5^ HLA-matched PBMCs for antigen presentation and 10 μg/ml T cell GAD. Epitope specificity of lines was examined using the same approach and individual peptides from a library of 115 overlapping peptides of GAD65 (20-mers overlapping by five amino acids; 10 μg/ml; Thermo Hybaid, Ulm, Germany;) were used as stimulation. Peptides eliciting a positive IL-13 response (spot number ≥ the inter-assay mean of the diluent +3 SD) were re-screened twice to confirm the result. In addition, as controls, Th1 and Th2 cell lines were generated using specific differentiating protocols and human cellXvivo Th1 and Th2 cell differentiation kits as instructed by the manufacturer (R & D systems, Abingdon, UK).

For the isolation of GAD-specific T cell clones, GAD-reactive T cell lines were stimulated with 10 μg/ml T cell GAD, labelled as above and CD4^+^IL-13^+^ cells were single-cell sorted into 96 well U-bottom plates containing 10^5^ irradiated mixed allogeneic PBMCs and PHA (4 μg/ml) in 200 μl complete X-VIVO media containing 5% (wt/vol.) human AB serum. The following day, the clones were supplemented with 10% (wt/vol.) TCGF and expanded in culture media containing decreasing concentrations of TCGF for 2 weeks. Clones and lines were expanded and maintained by alternate rounds of restimulation using PHA or T cell GAD and irradiated mixed allogeneic PBMCs.

In some experiments, intracellular cytokine staining was performed on GAD-specific clones and for comparison a haemagglutinin (HA)-peptide-specific flu clone following stimulation with phorbol 12-myristate 13-acetate (PMA)/ionomycin (Sigma Aldrich, Gillingham, UK) at 50 ng/ml and 1 μg/ml, respectively, or T cell GAD (10 μg/ml; Diamyd, Sweden) or GAD peptide (residues 555–567) or HA peptide (residues 306–318), accordingly (Thermo Hybaid, Ulm, Germany) at 10 μg/ml, followed by monensin and brefeldin A (Becton Dickinson) for 3 h at 37°C. The cells were then harvested and washed in PBS (Life Technologies, Paisley, UK) and stained with a live/dead marker (Aqua dead cell stain; ThermoFisher Scientific, Hemel Hempstead, UK) and following another wash in PBS, membrane staining was performed using mouse anti-human CD14 Pacific Blue (PB) (Thermo Fisher Scientific Cat. No. MHCD1428, RRID:AB_10373537), CD19 PB (Thermo Fisher Scientific Cat. No. MHCD1928, RRID:AB_10373689) (ThermoFisher Scientific), CD3 APC-H7 (Cat. No. 560275, RRID:AB_1645476, Becton Dickinson), CD4 PE-Cy5.5 (Cat. No. 560650, ThermoFisher Scientific) and CD8 PE-Cy7 (Cat. No. 344712 BioLegend) all made up according to manufacturer’s instructions for 20 min at 4°C according to manufacturers’ instructions. Cells were washed and fixed in CytoFix (Becton Dickinson) for 20 min at room temperature in the dark and subsequently permeabilised with permeabilisation solution (BioLegend) before intracellular staining using anti-human IL-13 APC (Cat. No. 501908), IL-4 FITC (Cat. No. 500807) (BioLegend) and anti-IFN-γ PE (Cat. No. 502508) (Becton Dickinson) at room temperature for 20 min. The cells were washed and resuspended for acquisition using a FACSCanto II flow cytometer (Becton Dickinson) and analysed using FlowJo v.10 (FlowJo, Ashland, OR, USA).

### T cell receptor single-cell sequencing and cytokine analysis of GAD-specific T cell lines and clones

T cell lines and clones were stimulated with 10 μg/ml T cell GAD or peptide or DMSO as a control for 48 h and IL-13 or IL-13/IFN-γ secreting cells captured using secretion/detection kits (Miltenyi Biotec, Bisley, UK) followed by sorting. Single-cell T cell receptor (TCR) sequencing and gene expression were performed as previously described [13, 14]. Sequencing results were annotated using the IMGT database [15], the CDR3A and CDR3B sequences extracted and compared across individual cells using KNIME 2.11.2 [16] and further analysed with R (version 3.4) (www.R-project.org/) and R package tcR [17] to determine identity with sequences in a repository of TCR sequences from circulating naive and central memory CD4^+^ T cells subsets (>2 × 10^8^ total sequences from 14 type 1 diabetes subjects and 17 healthy control individuals) [18].

For the analysis of gene expression, 10 μl of the first PCR product generated above were digested with exonuclease (ThermoFisher Scientific) and 1 μl was added to diethyl pyrocarbonate (DEPC)-treated water, 2× SYBR Premix Ex Taq (Takara, Saint-Germain-en-Laye, France) and forward and reverse primer mixes for each of the six genes (*IL13*, *GATA3*, *IL4*, *IFNG*, *TBX21* and *SRP14* as a control gene). Primer sequences listed in [13] and in ESM Table 3 were added and samples analysed in the ABI PRISM 7900HT sequence detection system qPCR Real-Time PCR machine (50°C for 2 min; 95°C for 10 min; [95°C for 15 s; 60°C for 1 min] × 40 cycles; 95°C for 15 s; 60°C for 15 s; 95°C for 15 s [ramp rate of 2%]). When the melting temperature of the amplified product was ±1°C of that of the positive control (cDNA from CD3^+^ cells), it was considered that the template of interest was present in the sample. Subsequently, C_t_ values were transformed into expression values (E) by subtracting them from 40 (E = 40 − C_t_), so higher values mean higher expression.

### Statistical analysis

The frequency of responses and responses examining fold changes in alum- and GAD-alum-treated participants were compared using Mann–Whitney *U* tests. ELISpot and cytokine responses at baseline vs post immunisation were analysed by Wilcoxon matched-pairs signed rank tests using GraphPad Prism software (version 8.3.1) Windows, GraphPad Software, San Diego, California USA, (www.graphpad.com). A *p* value of <0.05 was considered significant. Association between variables was assessed with Spearman’s rank correlation.

## Results

### GAD-specific Th2 responses are induced in GAD-alum-treated patients

**Fig. 1:**
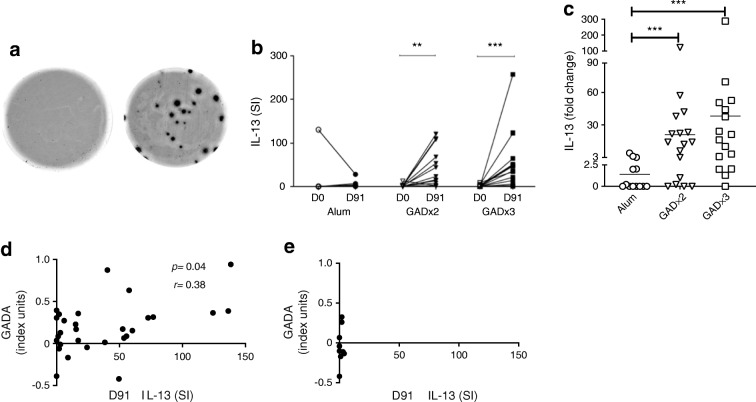
Increase in GAD-specific IL-13 responses in GAD-alum-treated participants. (**a**) Representative ELISpot images from a GAD-alum-treated patient at baseline and at day 91 post treatment. Spots represent IL-13 production by single CD4 T cells. IL-13 responses are unusual in type 1 diabetes but are induced after GAD-alum immunotherapy, as demonstrated in (**b**) showing increased frequency of GAD-specific IL-13 responses post vaccination with GAD-alum (twice [GAD×2] or three times [GAD×3], but not with alum alone (IL-13 stimulation index [SI] at day 0 [D0; baseline, first treatment] and day 91 [D91]). SI represents the number of spots after stimulation in vitro with recombinant human GAD (rhGAD65) divided by the number of spots after control stimulation. Differences between D0 and D91 for each of treatment group are shown using paired statistical comparisons using Mann–Whitney *U* tests (**p < 0.01; ***p < 0.001). (**c**) Responses can also be represented as fold change from baseline in participants receiving alum alone (circles) or GAD-alum twice (GAD×2) or three times (GAD×3). Statistical comparison of fold change is shown (***p < 0.001). (**d**, **e**) Induced GAD-specific IL-13 responses (shown as SI at day 91) correlate with change in anti-GAD autoantibodies (GADA) between baseline and day 91 in GAD-alum-treated participants (**d**) but not in those given alum alone (**e**). Note that post treatment GADA data are not available for the participant in the GAD×3 group with SI = 289 in (**b**) and (**c**). GADA levels were expressed as index units (antibody index value relative to standard serum)

Individuals receiving GAD-alum or alum were examined for IL-13 production by ELISpot using PBMC samples obtained at baseline and day 91 by operators blinded to the treatment group. In baseline samples from all the participants, GAD-specific IL-13 responses are present at a low frequency in new-onset type 1 diabetes, with nine out of 46 (20%) participants showing a response. GAD-alum immunotherapy resulted in a substantial increase in GAD-specific IL-13 responses at day 91 compared with baseline in participants receiving immunisations twice (*p* = 0.002) and three times (*p* = 0.0001) (Fig. 1a,b). In contrast, no change in GAD-specific IL-13 responses were seen in the participants who received placebo (alum alone). In participants treated with GAD-alum twice or three times, the fold change in IL-13 response from baseline was significantly greater compared with that in the placebo group (*p* = 0.003 and 0.0002, respectively) (Fig. 1c). A total of 12 out of 17 (71%; two GAD-alum injections) and 15 out of 16 (94%; three GAD-alum injections) had a more than twofold increase in IL-13 response to GAD between baseline and day 91, compared with five out of 13 (38%) participants treated with alum alone. Furthermore, the relationship between Th2 responsiveness and change in GADA titres was significant after GAD-alum treatment but was not significant after alum treatment (Fig. 1d,e).

There was no correlation between IL-13 responses and C-peptide AUC (data not shown). We also examined whether GAD-specific IL-13 responses were influenced by HLA haplotypes, specifically HLA-DR3 and HLA-DR4. Six of the GAD-alum-treated participants possessed the HLA-DR3/DR3 haplotype and four of these showed a GAD-specific IL-13 response (67%); 16 participants were positive for HLA-DR4 and of these 12 (75%), had a GAD-specific IL-13 response (DR3 vs DR4, *p* = 1); nine were HLA-DR3/DR4 positive and eight of these (89%) had a GAD-specific IL-13 response. In summary, there was no preferential association between HLA-DR haplotype and GAD-specific IL-13 responses.

Spontaneous cytokine secretion in response to 24 h of GAD65 stimulation in vitro was not observed in the baseline samples from all participants. However, at day 91 post immunisation in participants receiving GAD-alum twice there was a significant increase in secreted IL-5 (*p* < 0.05) and immunisation with GAD-alum three times induced a significant increase in secreted IL-13 (*p* < 0.01), IL-4 (*p* < 0.05), IL-5 (*p* < 0.01) and IL-17 (*p* < 0.05) (ESM Fig. 1). There was no change in the placebo (alum alone) group. There was also no increase in the immune-regulatory cytokine IL-10 post immunisation in GAD-alum-treated participants.

Overall, our findings indicate that GAD-alum immunotherapy is associated with a robust, adaptive immune response involving an expansion of the number of circulating GAD-specific T cells secreting the canonical Th2 cytokines IL-4, IL-5 and IL-13, directly linked to changes in the GAD-specific humoral response.

### T cell responses generated after GAD-alum immunisation target multiple GAD65 epitopes

**Fig. 2:**
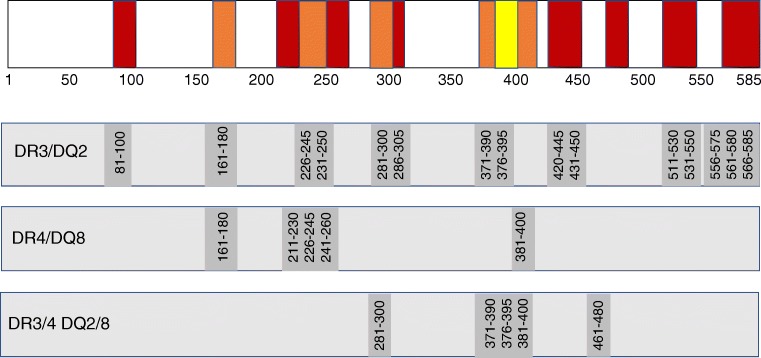
Immunogenic regions of GAD65. Regions were mapped by screening overlapping GAD65 peptides with GAD-specific T cell lines generated using PBMCs from participants immunised with GAD-alum and tested for IL-13 response by ELISpot. Lines were generated from participants homozygous for HLA-DR3/DQ2 (DRB1*0301/DQA1*0501-DQB1*0201), homozygous for HLA-DR4/DQ8 (DRB1*0404/DQA1*0301 DQB1*0302) and heterozygous for HLA-DR3/4 DQ2/8. Epitope regions are shown in red when they are observed in only one line, orange when observed in two lines and yellow when multiple lines show reactivity

**Fig. 3:**
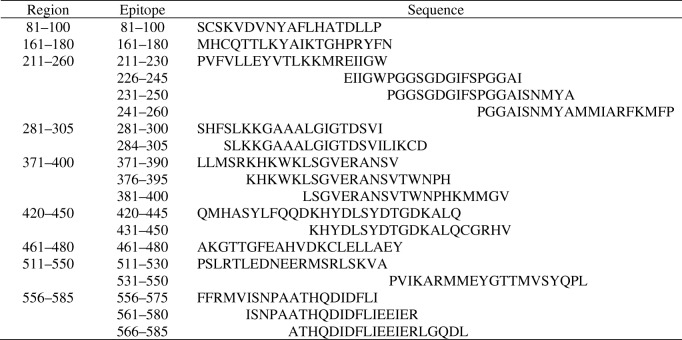
GAD epitopes identified by screening overlapping peptides for IL-13 responses

To characterise these responses further and to facilitate studies at single-cell resolution, we examined the epitope specificity of type 2 responses to GAD. T cell lines were generated from four GAD-alum-immunised participants; two lines were generated from participants homozygous for the HLA-DRB1*0301/DQA1*0501/DQB1*0201 genotype, one from an individual homozygous for HLA-DRB1*0404/DQA1*0301/DQB1*0302 and a further line from a heterozygous individual (HLA-DRB1*0301/DQA1*0501/DQB1*0201; DRB1*0401/DQA1*0301/DQB1*0302). T cell lines were used to screen peptides overlapping the entire GAD65 sequence by IL-13 ELISpot assay. For each line, multiple epitopes were identified throughout the GAD65 sequence (Figs. 2 and 3).

For the HLA-DRB1*0301/DQA1*0501/DQB1*0201 participant lines, 15 peptides were identified that elicited an IL-13 response, nine of which were nested around adjacent overlapping sequences (GAD226-245, GAD231-250, GAD281-300, GAD286-305, GAD371-390, GAD376-395, GAD556-575, GAD561-580, GAD566-585) (Fig. 2) and six represented single sequences (GAD81-100, GAD161-180, GAD420-445, GAD431-450, GAD511-530 and GAD531-550).

Five peptides were recognised by the HLA-DRB1*0404/DQA1*0301/DQB1*0302 participant line encompassing peptides GAD161-180, GAD211-230, GAD226-245, GAD241-260 and GAD381-400 (Fig. 2). Two of these peptides (GAD161-180 and GAD226-245) appear promiscuous as they were also targeted by the HLA-DRB1*0301/DQA1*0501/DQB1*0201 participant line.

For the heterozygous HLA-DR3/DR4 participant line, IL-13 responses were detected against five peptides, three of which were adjacent overlapping sequences (GAD371-390, GAD376-395 and GAD381-400) and single peptides GAD281-300 and GAD461-480.

To summarise these findings, induced Th2 responses to GAD65 target multiple regions across the molecule, some of which overlap in individuals with different HLA genotypes.

### T cells generated after GAD-alum immunisation display a bifunctional Th1/Th2 phenotype

The ELISpot and cytokine secretion analyses show that GAD-alum immunisation generates a GAD-specific Th2 response. We and others have previously reported that GAD-specific Th1 responses are a feature of the natural history of type 1 diabetes [12, 19, 20]. Since the proposed mechanism of action of GAD-alum is immune diversion of autoreactive Th1 to Th2 responses, we next examined the fate of anti-GAD Th1 responses present at baseline and their relationship to the development of treatment-induced anti-GAD Th2 responses, using a FluoroSpot assay that simultaneously detects secretion of both IFN-γ and IL-13 on a single-cell-specific basis.

**Fig. 4:**
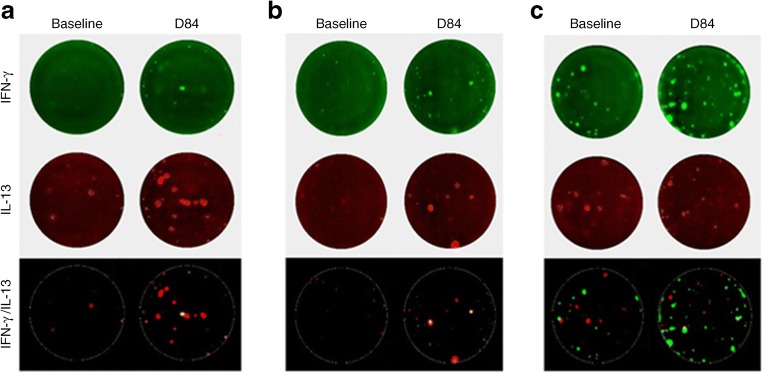
Examples of GAD-specific bifunctional Th1/Th2 cells induced upon GAD-alum immunisation. Representative data from two GAD-alum-vaccinated participants showing responses to GAD65 peptides (**a**) 231–250 and (**b**) 556–575 at baseline and day 84 (D84) post immunisation analysed by dual IL-13/IFN-γ indirect FluoroSpot. In the merged image panels, dual-cytokine-secreting cells appear yellow. (**c**) Baseline and day 84 responses to the recall antigen Pediacel for comparison. IFN-γ responses are shown in green, IL-13 in red and bifunctional Th1/Th2 responses are yellow

**Fig. 5:**
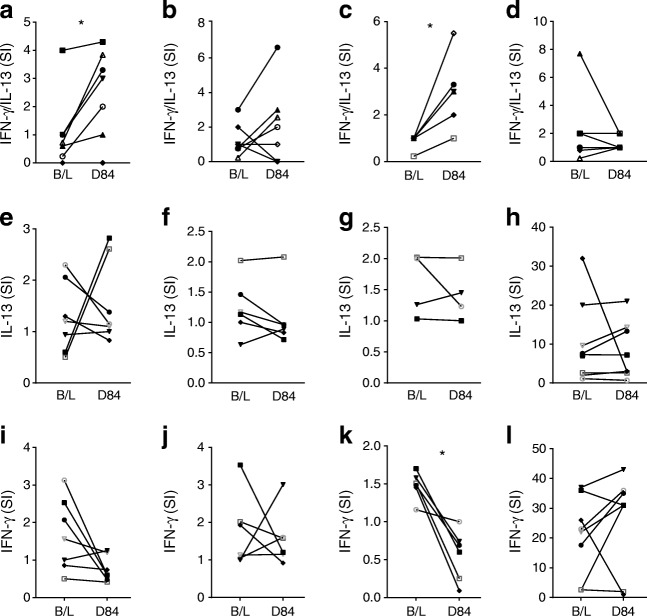
GAD-specific bifunctional cells are induced upon GAD-alum immunisation. IFN-γ and IL-13 responses to GAD epitopes: GAD226-245 (**a**, **e**, **i**), GAD231-250 (**b**, **f**, **j**), GAD556-575 (**c**, **g**, **k**) and Pediacel (**d**, **h**, **l**) were analysed by dual IL-13/IFN-γ indirect FluoroSpot in nine participants immunised with GAD-alum at baseline (B/L) and at day 84 (D84). Bifunctional Th1/Th2 responses are shown in (**a**–**d**), single IL-13 in (**e**–**f**) and IFN-γ responses in (**i**–**l**). Each symbol represents a different immunised participant. The comparison between responses at baseline and D84 post immunisation were analysed by Wilcoxon matched-pairs signed rank tests (*p < 0.05)

We confirmed previous findings, namely that a subset of individuals (31 out of 71; 44%) tested at onset of type 1 diabetes is characterised by the presence of GAD-specific IFN-γ-secreting T cells, which are revealed after stimulation ex vivo with GAD65 (ESM Fig. 2). However, the most striking observation is that post GAD-alum immunisation, there is a population of IL-13-secreting T cells that also produce IFN-γ which is absent from baseline samples (Fig. 4). IL-13^+^/IFN-γ^+^ cells specific for GAD are significantly expanded in GAD-alum post-immunisation samples compared with baseline samples (e.g. for the peptides GAD226-245 and GAD556-575, *p* = 0.02 and *p* = 0.03, respectively) but not in placebo (alum) post-immunisation samples (Fig. 5; ESM Figs. 3, 4). Dual-positive cells in response to the recall antigen Pediacel did not change in number during the study. In contrast, the magnitude of single GAD-specific IFN-γ or IL-13 responses remained unchanged (GAD226-245) or declined significantly (GAD556-575) compared with baseline. In addition, GAD-specific IFN-γ^+^/IL-13^+^-co-secreting CD4 T cells represent a stable phenotype, induced by GAD-alum, since they remained detectable in samples tested up to 12 months after immunisation (*n* = 4 individuals) (ESM Fig. 5). While these peptide-based studies provide strong confirmatory evidence of antigen specificity, in concert with the experiments using whole GAD65, it is possible that some of the responses reflect a degree of cross-reactivity, perhaps as a result of peptides binding to HLA molecules in unconventional registers.

### GAD-specific T cell clones display bifunctionality

**Fig. 6:**
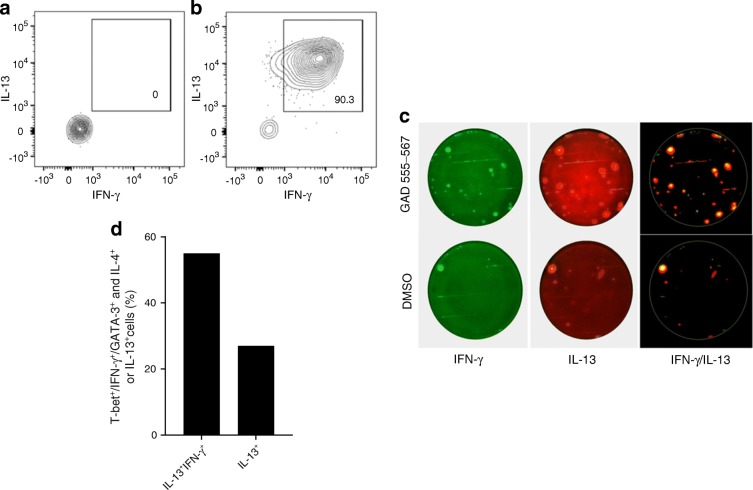
GAD-specific CD4 T cells induced by GAD-alum immunisation are bifunctional. (**a**) Representative GAD-specific CD4 T cell clone stimulated with diluent DMSO or (**b**) PMA/ionomycin for 3 h and stained for intracellular expression of IL-13 and IFN-γ shows the clone’s bifunctional potential. (**c**) GAD peptide-specific clone exhibits a bifunctionality when stimulated with cognate GAD65 555–567 peptide in vitro and analysed by dual-cytokine FluoroSpot. The control condition is stimulation with DMSO (the peptide diluent). The Th1/Th2 phenotype (IFN-γ^+^/IL-13^+^) is shown in yellow. IFN-γ responses are green and IL-13 are red (representative data from six clones). (**d**) Expression of cytokine and transcription factor mRNA by single sorted clone/line cells detected by PCR shows that a majority of GAD-alum induced bifunctional CD4 T cells co-express Th1 and Th2 differentiation markers *TBX21* (encoding T-bet), *IFNG* and *GATA3* as well as *IL4* and/or *IL13*

To examine the nature of GAD-specific IL-13-secreting T cells induced upon GAD-alum immunisation at single-cell resolution we generated 22 GAD-specific T cell clones from a GAD-specific T cell line from an immunised individual using a strategy that specifically enriches for CD4^+^IL-13^+^ cells. For 21 out of 22 clones we observed that IL-13 secretion was accompanied by IFN-γ production following PMA/ionomycin stimulation in vitro (Fig. 6a). A median of 15% (range 2–90%) of cells in each of the clone populations co-produced both IL-13 and IFN-γ in response to this polyclonal stimulus (not shown), and dual-cytokine production (IL-13 and IFN-γ) was also seen following stimulation with cognate peptide in a dual-colour FluoroSpot assay (Fig. 6c). In addition, we examined expression of canonical markers of Th1 and Th2 differentiation at single-cell resolution at the transcriptional level (canonical Th1 transcription factor *TBX21* and cytokine *IFNG*; canonical Th2 transcription factor *GATA3* and cytokines *IL4*, *IL13*). The majority of GAD65-stimulated CD4 T cells from specific lines and clones exhibited a hybrid phenotype, expressing a combination of *TBX21*, *IFNG* and *GATA3*, as well as *IL4* and/or *IL13* (Fig. 6d); in contrast this hybrid phenotype was observed in 2% (1/41) and no (0/42) pure Th1 and Th2 lines, respectively (not shown).

### Th1/Th2 bifunctional T cells generated after GAD-alum immunisation originate from naive and memory compartments

**Fig. 7:**
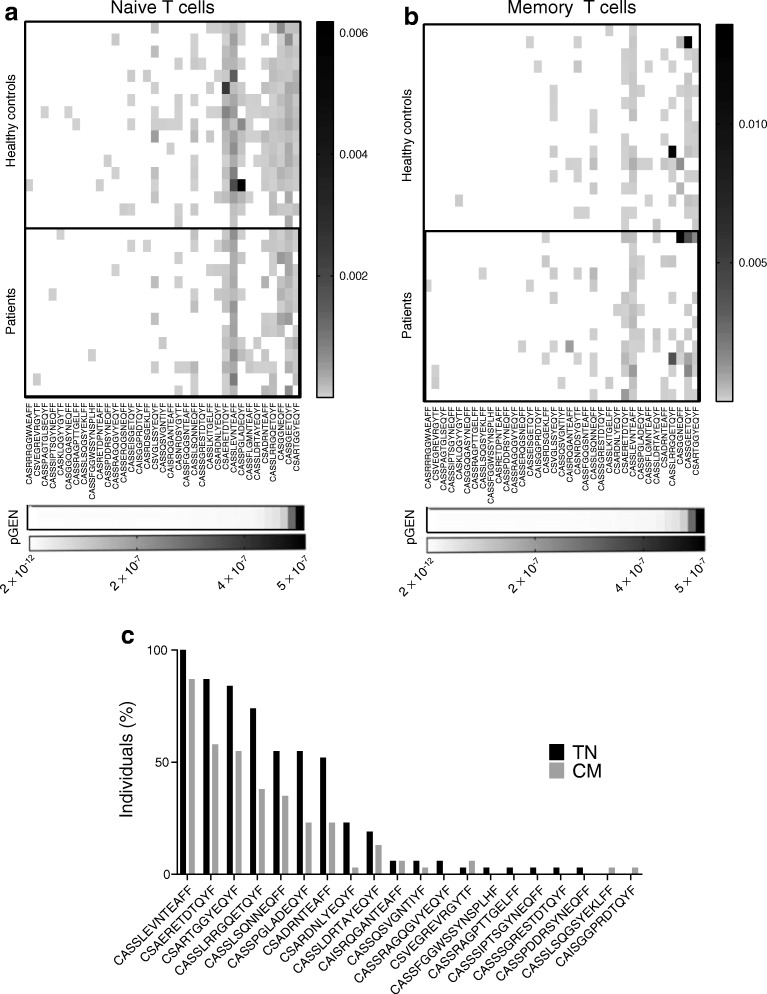
T cell receptors of Th1/Th2 bifunctional cells. Th1/Th2 bifunctional cells induced upon GAD-alum immunisation have the potential to arise after priming from naive cells and/or expansion from GAD-specific central memory CD4 T cells. TCRB CDR3s from bifunctional cells from GAD-specific T cell lines generated post GAD-alum immunisation were sequenced; these were then compared against TCRB CDR3s bulk sequenced from naive and central memory CD4^+^ T cell subsets from participants with type 1 diabetes (*n* = 14) and healthy controls (*n* = 17) (true naive, TN; central memory, CM) [18]. The CDR3s from bifunctional Th1/Th2 cells are identifiable in both (**a**) naive and (**b**) central memory cells. The probability of generation (pGEN) values are shown on the bottom of the panels: values ranging from 10^−7^ to 10^−10^ indicate a high chance of a TCR being generated and therefore likely to be public. (**c**) The prevalence of clonotypes amongst all participants

We hypothesised that GAD-specific bifunctional Th1/Th2 cells could originate in two ways: following GAD-alum immunisation, naive T cells could be activated de novo under conditions that favour bifunctionality; or pre-existing GAD-specific memory T cells could be expanded under conditions that favour deviation to bifunctionality. To begin to address this, we sequenced T cell receptor β-chain (TCRB) CDR3 regions of GAD-specific bifunctional T cells obtained from two of the T cell lines (one donor HLA-DRB1*0301/DQA1*0501/DQB1*0201 genotype, one HLA-DRB1*0404/DQA1*0301/DQB1*0302) and matched these against a repository of TCRB CDR3 sequences from circulating naive, and central memory CD4^+^ T cell subsets that we obtained previously from a separate cohort of non-GAD immunised, new-onset type 1 diabetes individuals and HLA-matched, non-diabetic healthy individuals [18]. A total of 61 discrete TCRB CDR3 region sequences were obtained from a total of 271 sorted GAD-specific bifunctional T cells. Of these 61, 20 had amino acid sequences that matched identically to sequences in our naive and central memory CD4^+^ T cell repository. Of these, 12 out of 20 were found in naive and central memory cells, six out of 20 were exclusively in naive cells and two out of 20 were exclusively in the central memory CD4 T cell populations in both participants with type 1 diabetes and healthy individuals (Fig. 7a,b). One TCRB CDR3 region (CASSLEVNTEAFF) was present in the naive pool in every individual screened, indicating that this is a highly public clonotype. The very public nature of the CASSLEVNTEAFF clonotype has been highlighted by others both at the amino acid [21, 22] and nucleotide level (tgtgccagcagtttggaggtgaacactgaagctttcttt) (http://clonesearch.jdrfnpod.org/). The mean frequency of central memory clonotypes that shared TCRB CDR3s with GAD-specific T cells is higher in individuals with type 1 diabetes than in healthy control individuals but not significant (*p* = 0.052) (data not shown).

With the caveat that we have only examined one chain of the TCR, these data suggest that T cells with the potential to recognise GAD epitopes in immunised patients are expanded from a highly public TCRB repertoire that may be present in both the naive and central memory cell pools in both individuals with type 1 diabetes and healthy individuals.

## Discussion

GAD-alum immunotherapy has the stated aim of subverting T cell-mediated autoimmune destruction of islet beta cells by redirecting a predominant (‘pathogenic’) Th1 response towards a Th2 response. While immunological studies, including ours, indicate that Th2-like responses are indeed detectable after GAD-alum immunisation [7, 23], this change has not been unequivocally shown to translate into preservation of beta cell function or clinical benefit [4, 24]. In the present study we explored this disconnect between immunological and clinical efficacies. Through a detailed molecular and cellular examination of GAD-specific CD4 T cells induced by GAD-alum immunisation we demonstrate that a majority of the responder clones harbour features of both Th1 and Th2 cells and can thus be considered bifunctional. We speculate that this phenotype lacks immune-modulating properties and hence may not be capable of modifying autoimmunity in a clinically favourable way.

Bifunctional cells that share lineage and phenotypic features of two canonical CD4 T cell differentiation pathways have been described previously in autoimmune disease and in other settings. Th1/Th2 bifunctional cells were reported in humans in progressive multifocal leucoencephalopathy immune reconstitution inflammatory syndrome (PML-IRIS), a complication of monoclonal antibody therapy in multiple sclerosis. CD4 T cells isolated from brain tissue were found to secrete both IL-4 and IFN-γ and express GATA-3 and T-bet (encoded by *TBX21*) [25]. Our report of GAD-specific Th1/Th2 bifunctional CD4 T cells both upholds and extends these observations, since we not only confirm the presence of these cells in the circulation, but we also identify them as autoreactive, which to our knowledge has not been reported before. Other studies have suggested bifunctionality of cells co-expressing IFN-γ and IL-17 in humans with inflammatory bowel disease and systemic lupus erythematosus [26, 27]. We established the presence of bifunctional IL-4/IL-13^+^IFN-γ^+^ cells induced upon GAD-alum immunisation at single-cell resolution for both protein production and gene expression using assays that measure responses directly ex vivo. Th1/Th2 cells were present in approximately two-thirds of participants treated with GAD-alum. Moreover, we demonstrated that GAD-specific CD4 T cell clones and lines derived from treated participants after expansion in vitro co-produce both IL-13 and IFN-γ and express the Th2 cytokine IL-4 as well as the canonical transcription factors (T-bet and GATA-3) for Th1 and Th2 cells, respectively. Participants treated with alum alone rarely presented an IL-13 response. Once induced by immunisation, bifunctional GAD-specific Th1/Th2 CD4 T cells are robustly stable for up to 12 months, a feature that has also been reported by others [28]. Interestingly, increased GAD-specific IFN-γ and IL-4 responses by ELISpot have been reported previously in GAD-alum-treated participants [29], but in this case it was not established whether these were produced simultaneously by the same cell.

Bifunctional Th1/Th2 CD4 T cells may arise in vivo during infections and display functional properties that are quantitatively intermediate between Th1 and Th2 cells [30]. As such, they are considered to be attenuated in immunological potency, cause less immunopathology, and have a self-limiting role perhaps designed to limit excessive inflammation [30]. A similar self-limiting function has been assigned to skin-resident Th17 cells that are poised to adopt a Th2 phenotype in cases of tissue injury or impaired regulation which licenses type 2 immunity and thereby promotes tissue repair [31]. Collectively, as our observations based on stable cytokine secretion indicate, bifunctional CD4 T cells represent a defined subpopulation of differentiated cells rather than an intermediate stage.

How a population of Th1/Th2 cells arises upon GAD-alum immunisation is open to speculation. As demonstrated in the present study and previously reported by others [29, 32], multiple cytokines are detected in the supernatants of GAD-stimulated cultures from GAD-alum-immunised participants, and it is possible that if these conditions are reproduced in tissues in vivo upon immunisation, this distinctive cytokine milieu is a potent imprinting mechanism, either redirecting pre-existing GAD-specific Th1 or, more rarely Th2 cells; or inducing bifunctionality de novo. Of note, we did not find any evidence that the bifunctional response was focused onto any particular immunogenic regions of the GAD65 molecule, but this may be worthy of further analysis and fine-mapping of responses at the clonal level.

Finally, we attempted to address the question as to whether expanded bifunctional Th1/Th2 cells derive from the antigen-inexperienced or naive CD4 T cell pool. By searching for TCRB CDR3 sequences from induced bifunctional CD4 T cell clone responses in a repository of TCR sequences from circulating naive and central memory CD4^+^ T cell subsets from individuals with new-onset type 1 diabetes and healthy individuals we were able to show that numerous GAD-specific clonotype signatures are public, being present in multiple people; and not disease-specific, being present in both those with diabetes and the healthy control individuals. We observed that TCRB CDR3 clonotypes were present at a greater frequency in the central memory pool in individuals with type 1 diabetes, suggesting that they are preferentially expanded as part of the disease process.

Thus, CD4 T cells recognising GAD through a distinctive, bifunctional differentiation pathway are expanded numerically post immunisation on the background of a potentially facilitating, highly public and disease-agnostic TCRB repertoire. The relevance of this process to the equivocal clinical outcomes seen in proof-of-concept efficacy studies is unclear, but it is reasonable to hypothesise that bifunctional cells are attenuated in any form of counter-regulatory function and thus do not sufficiently divert the pathogenic autoimmunity responsible for type 1 diabetes progression.

## Electronic supplementary material

ESM 1 (PDF 447 kb)

## Data Availability

The data are available upon request from the authors.

## Abbreviations

Alum: Aluminium hydroxide
ASI: Antigen-specific immunotherapy
GADA: GAD autoantibodies
HA: Haemagglutinin
PBMC: Peripheral blood mononuclear cell
PE: Phycoerythrin
PHA: Phytohaemagglutinin
PMA: Phorbol 12-myristate 13-acetate
Th: T helper cell
TCGF: T Cell growth factor
TCR: T cell receptor
TCRB: T cell receptor β-chain
