# Supplementary material for: GAD-alum immunotherapy in type 1 diabetes expands bifunctional Th1/Th2 autoreactive CD4 T cells
Source: Diabetologia. 2020 Apr 4;63(6):1186–98. doi: 10.1007/s00125-020-05130-7 (PMC7228993; doi:10.1007/s00125-020-05130-7)
Supplement: Supplementary file 1 — (PDF 447 kb) [file 125_2020_5130_MOESM1_ESM.pdf]

ESM Table 1. Comparison of participant characteristics in our mechanistic study and the original TrialNet intervention study, TN08.

	**Mechanistic Sub-study (n=46)**			**Complete TrialNet Study (n=145)**		
	Alum	GAD-Alum x2	GAD-Alum x3	Alum	GAD-Alum x2	GAD-Alum x3
Age (years):						
Mean (SD)	16.2 (9.5)	16.9 (10.5)	19 (10.6)	16.6 (9.23)	14.9 (8.72)	17.9 (10.4)
Median	12 (8-42)	14 (4-45)	16 (6-42)	14.5 (4-45)	14.0 (3-45)	15.5 (3-44)
Female sex	4 (31%)	10 (59%)	6 (38%)	19 (40%)	31 (63%)	14 (29%)
White race*	12 (92%)	15 (88%)	15 (94%)	40 (85%)	45 (94%)	43 (91%)
Non-Hispanic ethnic origin	11 (85%)	16 (94%)	14 (88%)	48 (100%)	49 (100%)	48 (100%)
Autoantibodies:						
1	1 (8%)	0	1 (6%)	1 (2%)	0	3 (6%)
2	1 (8%)	2 (12%)	4 (25%)	10 (21%)	10 (20%)	10 (21%)
3	8 (62%)	10 (59%)	6 (38%)	24 (50%)	23 (47%)	14 (29%)
4	3 (23%)	5 (29%)	5 (31%)	13 (27%)	16 (33%)	21 (44%)
GADA titre (index units)	0.451 (0.337)	0.492 (0.408)	0.140 (0.134)	0·336 (0·307)	0·349 (0·381)	0·230 (0·224)
Diabetes-associated HLA present*						
DR3 & DR4	4 (31%)	5 (29%)	4 (25%)	14 (30%)	7 (14%)	13 (27%)
DR3 only	3 (23%)	4 (24%)	2 (12.5%)	8 (17%)	13 (27%)	15 (31%)
DR4 only	4 (31%)	8 (47%)	8 (50%)	20 (43%)	21 (43%)	13 (27%)
Neither	2 (15%)	0	2 (12.5%)	5 (11%)	8 (16%)	7 (15%)

*Descriptive statistics excludes some subjects (2 – race not reported)

ESM Table 2. Characteristics of non-trial subjects with type 1 diabetes included in the mechanistic study.

	T1D patients (n=71)
Age (years):<br><br>Mean (SD)<br>Median	 <br>28.7 (6.78)<br>29 (18-46)
Female sex	24 (34%)
Mean Duration of diabetes (months)	6 (0.25-14.5)
Diabetes-associated HLA present*:<br><br>DR3 & DR4<br>DR3 only<br>DR4 only<br>Neither	 <br>18 (26%)<br>10 (15%)<br>34 (50%)<br>6 (9%)

*Descriptive statistics excludes some subjects (3: HLA alleles not tested)

ESM Table 3: Oligos used for 1st PCR and qPCR

Oligo name	Sequence
1st PCR	
INFg-1_3’	TGGATGCTCTGGTCATCTTT
INFg-1_5’	CTGTTACTGCCAGGACCCAT
IL4_1_3’	CTCTGGTTGGCTTCCTTCAC
IL4_1-5’	TGCCTCCAAGAACACAACTG
IL-13-1_3’	TTTACAAACTGGGCCACCTC
IL-13-1_5’	GGTCAACATCACCCAGAACC
T-bet_1-3’	ATCTCCCCCAAGGAATTGAC
T-bet_2-5'	CCGTGACTGCCTACCAGAAT
GATA3_1-3'	TTGGAGAAGGGGCTGAGAT
GATA3_1-5'	CCGCCCTACTACGGAAACTC
SRP14_3’	GCTGCTGCTTTGGTCTTCTT
SRP14_5’	TATGACGGTCGAACCAAACC
qPCR	
INFg-1_3’	TGGATGCTCTGGTCATCTTT
INFg-2_5’	GGTCATTCAGATGTAGCGGA
IL4_1_3’	CTCTGGTTGGCTTCCTTCAC
IL4_2-5’	GGCAGTTCTACAGCCACCAT
IL-13-1_3’	TTTACAAACTGGGCCACCTC
IL-13_2_5’	GTACTGTGCAGCCCTGGAAT
T-bet_1-3’	ATCTCCCCCAAGGAATTGAC
T-bet_2-5'	CCGTGACTGCCTACCAGAAT
GATA3_1-3'	TTGGAGAAGGGGCTGAGAT
GATA3_1-5'	CCGCCCTACTACGGAAACTC
SRP14_3’	GCTGCTGCTTTGGTCTTCTT
SRP14_5’-2	TACTGTGGAGGGCTTTGAGC

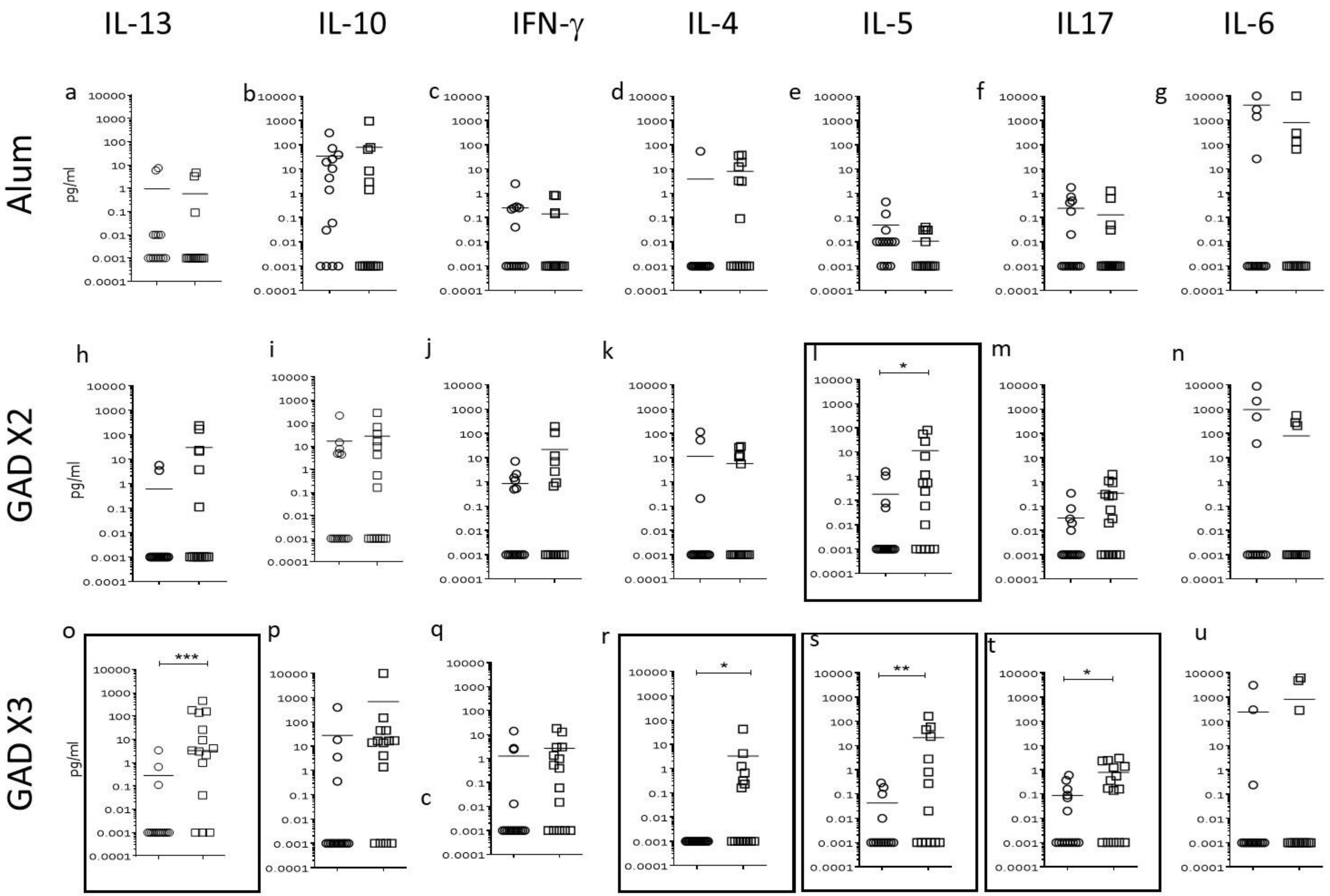

ESM Fig 1.: Th2 cytokines dominate T cell responses detected *in vitro* in response to stimulation with GAD65 in GAD-Alum treated patients. GAD-specific cytokine responses (log10) are shown at baseline (circles) and at day 91 (squares) for all treatment groups. Panels highlighted in black indicate significant changes upon vaccination compared to baseline samples (*p<0.05, ** p<0.01, ***p<0.001).

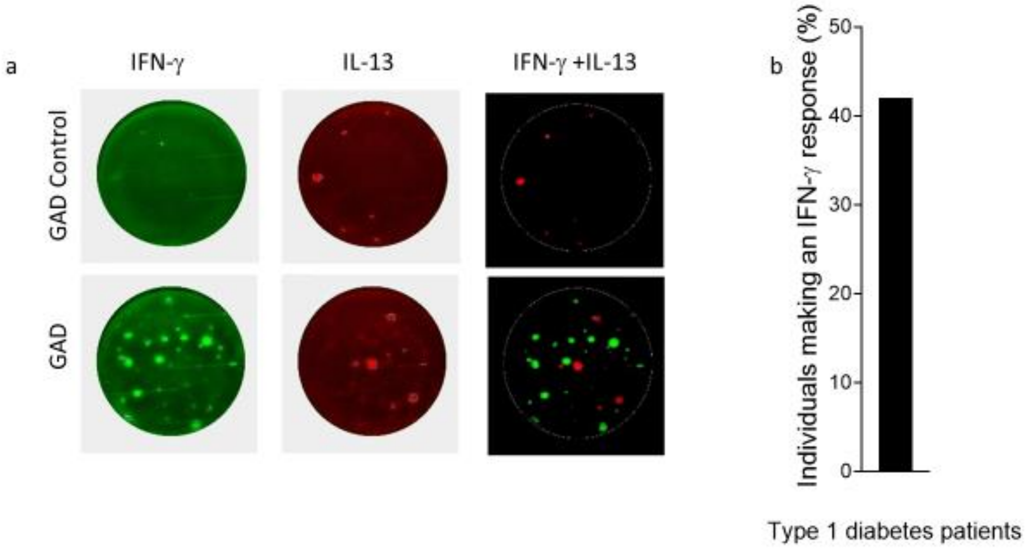

ESM Fig 2: IFN-γ and IL13 responses to GAD65 antigen (or antigen control) in non-TN08 patients with recent-onset type 1 diabetes. Panel (a) shows the dual-colour fluorospot response of a representative patient in which both IFN-γ (green spots) and IL-13 (red) responses can be detected in spontaneous disease and in the absence of GAD-Alum immunization. Of note, in the merged image there are no IFN-γ+IL-13+ dual-expressing cells, which would appear yellow. Overall, (b) after testing 74 non-trial patients with recent-onset type 1 diabetes, the prevalence of spontaneous IFN-γ responses to GAD65 is 42%.

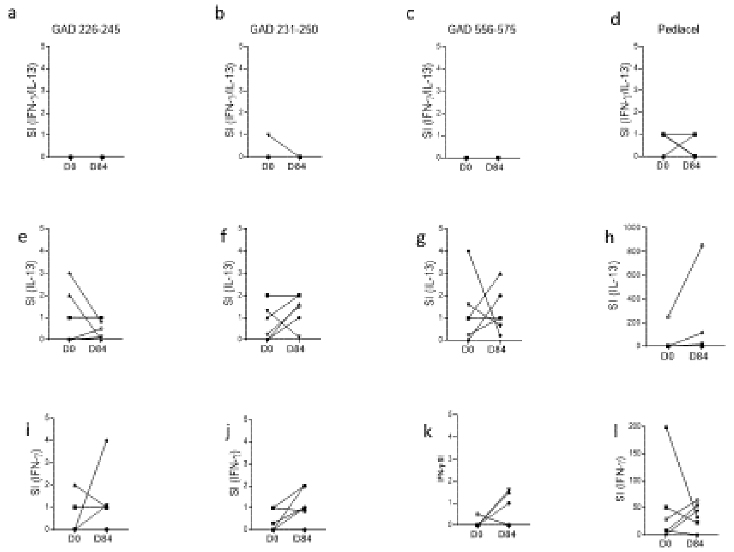

ESM Fig 3: IFN-γ and IL-13 responses to GAD epitopes and Pediacel were analysed in 6 participants immunised with Alum at baseline and at day 84; bi-functional Th1/Th2 responses are shown in the top panel, single IL-13 in the middle panel and IFN-γ responses in the bottom panel. Each symbol represents a different immunized subject.

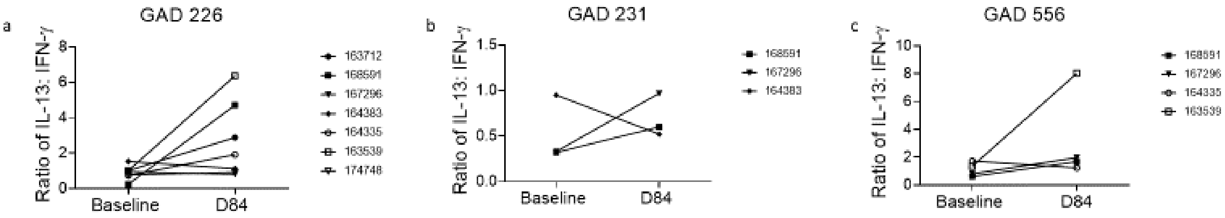

EMS Fig 4: GAD-specific bi-functional cells are induced upon GAD-Alum immunization. The ratio of IL13 to IFN-γ responses in baseline and D84 post-treatment samples.

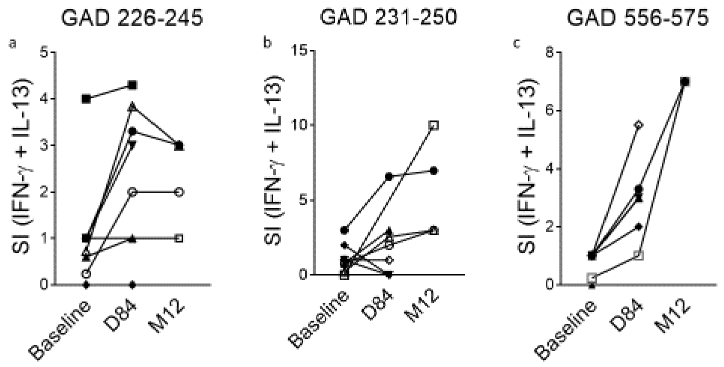

ESM Fig 5Bi-functional Th1/Th2 responses are stable for up to 12 months. Responses were analysed in 4 patients 12 months post-immunisation with GAD-Alum; Each symbol represents a different immunized subject.
